# Hepatitis B serologic survey and review of immunization records of children, adolescents and adults in Fiji, 2008–2009

**DOI:** 10.1186/s12985-015-0267-7

**Published:** 2015-03-03

**Authors:** Tatsuhiko Tsukakoshi, Josaia Samuela, Eric V Rafai, Uraia Rabuatoka, Sumihisa Honda, Yasuhiko Kamiya, Corazon C Buerano, Kouichi Morita

**Affiliations:** Department of Virology, Institute of Tropical Medicine, Nagasaki University, 1-12-4Sakamoto, Nagasaki City, 852-8523 Japan; Ministry of Health and Medical Services, Suva, Fiji; Public Health Laboratory, Fiji Centre for Communicable Disease Control, Suva, Fiji; Department of Nursing, Graduate School of Biomedical Science, Nagasaki University, 1-12-4 Sakamoto, Nagasaki, 852-8523 Japan; Graduate School of International Health Development, Nagasaki University, 1-12-4 Sakamoto, Nagasaki, 852-8523 Japan

**Keywords:** Hepatitis B immunization program, EPI, Immunization coverage, Seroprevalence

## Abstract

**Background:**

In Fiji, hepatitis B (HB) vaccine was introduced into childhood immunization program in 1989 and has been administered as a pentavalent since 2006. This study aimed to: (i) survey and examine the extent to which HB infection continue to occur in children, adolescents and adults in Fiji, and (ii) determine HB coverage rates and timeliness of vaccine administration to children.

**Methods:**

Serum samples of children, adolescents and adults (aged 6 months to <5 years, 16–20 years, and 21–49 years, respectively) collected between 2008–2009 were tested for serologic markers of HB virus infection namely, HB surface antigen (HBsAg), anti-HBs and anti-HB core antigen (anti-HBc). Health record card of each child was reviewed.

**Results:**

None of the participating children (0/432) was positive for HBsAg. Overall prevalence of HBsAg among adolescents and adults was 5.6% (7/124) and 3.2% (12/370), respectively. High prevalence (98.1%) of anti-HBs was observed in children. An estimated 17.4% of adolescents and adults had evidence of past HBV infection (anti-HBc positive), of which 87.2% recovered from infection but the remaining 12.8% developed chronic infection. Percentage of children who completed at least 3 doses of HB immunization was 99.3%, and who received them on schedule was 58.5%.

**Conclusion:**

Although sample populations for this study is less robust compared to 1998, the prevalence of HBsAg and anti-HBc in children and adults before and after the implementation of the immunization program is much lower. The findings are a positive step in showing that Fiji’s HB vaccine control program is achieving its objectives.

## Introduction

Hepatitis B virus (HBV) is a DNA virus and humans are the only known natural host. HBV infection can lead to a person’s premature death from cirrhosis, liver failure and liver cancer. HBV is transmissible through several routes: (i) percutaneous - injecting drug use, exposure to contaminated blood or body fluid; (ii) sexual - heterosexual or male homosexual activity; (iii) vertical - from mother to infant; and (iv) horizontal - between children and household contacts through skin lesions or sharing of blood-contaminated toothbrushes and razors [1,2].

Children aged between one and five years when infected with HBV have a 20-50% chance of developing chronic infection [3]. This may progress to hepatocellular carcinoma later in the adult life at a rate of 5% per decade, which is 100 to 300 times the rate observed among uninfected people in the general population [4]. Because the risk of chronic infection is inversely correlated with age, people who are infected as children bear a large burden in terms of morbidity and mortality [2].

Immunization against HBV is the most effective measure to prevent HBV infection. In Fiji, hepatitis B (HB) vaccine was introduced into the childhood immunization program in 1989 [5]. The vaccine administered since 2006 is a monovalent vaccine for the birthdose and a pentavalent (DTP-HepB-Hib) vaccine for the succeeding three doses [6]. A segment of the population under the age of 20 years was 38.5% in 2007 in Fiji, and an estimated 40% of the total population would have been covered by HB immunization in 2009 [7].

A seroprevalence study conducted among pre-school immunized children in Fiji in 1998 revealed that the prevalence of hepatitis B surface antigen (HBsAg), antibody to hepatitis B surface antigen (anti-HBs) and antibody to hepatitis core antigen (anti-HBc) was 0.7%, 77.0%, and 5.3%, respectively [8]. Among unimmunized mothers the prevalence was 6.6%, 57.9% and 67.2%, respectively. The present study was initiated as part of a technical cooperation titled “Project for strengthening expanded immunization program in the Pacific region” funded by the Japan International Cooperation Agency (JICA), an official overseas aid agency under the Japanese government. The project was characterized by activities in line with the PIPS (Pacific Immunization Programme Strengthening) strategy, which is the regional framework that enables PIPS international partners, including JICA, WHO, UNICEF, CDC and other agencies to share the vision of cooperation and support to immunization programs in the Pacific Island Countries.

The information yield by this study defines the magnitude of trends of HB infection status among different age groups and presents an impact of the HB immunization program performance.

## Results

### Hepatitis B serologic survey

**Figure 1:**
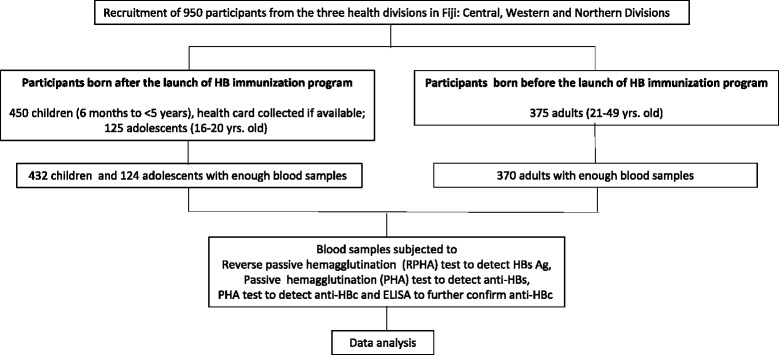
**Flow-chart of the study.**

A total of 950 participants were recruited from the three health divisions of Fiji (Central, Western and Northern Divisions, Figure 1) but only 926 recruits (432 out of 450 children, 124 out of 125 adolescents and 370 out of 375 adults, Table 1) were included in the serologic survey. Eighteen children were excluded from the survey for any of the following reasons: small volume of blood obtained, unsuccessful venipuncture and a very sick child. An adolescent and five adults were later excluded from the study because they did not show up at the designated clinic at the appointed time and date or because their bad physical condition made them unfit for blood sampling.

**Table 1:** **Number of participating children, adolescents, and adults (born after or before the launch of immunization program) under each health service division**

No. of participants tested*/total number of recruited participants (%)				
	**Born after launch of HBB immunization program**		**Born before launch of HBB immunization program**	
**Division**	**Children (6 mos. to <5 yrs. old)**	**Adolescents (16 to 20 yrs. old)**	**Adults (21 to 49 yrs. old)**	**Total**
Central	193/204 (94.6%)	50/50 (100%)	149/150 (99.3%)	392/404 (97.0%)
Western	168/175 (96.0%)	49/50 (98.0%)	148/150 (98.7%)	365/375 (97.6%)
Northern	71/71 (100%)	25/25 (100%)	73/75 (97.3%)	169/171 (98.85)
Total	432/450 (96.0%)	124/125 (99.2%)	370/375 (98.7%)	926/950 (97.5%)

*Refers to the number of participants with the blood samples subjected to serological tests.

Four hundred thirty two children were negative for HBsAg; while adolescents and adults had a 5.6% (7/124) and 3.2% (12/370) prevalence, respectively and a combined prevalence of 3.84% (19/494) (Table 2). The prevalence of HBsAg was highest in adolescents (5.6%) but it was not statistically significant (*P* = 0.4253, chi-squared test) from that of the other two populations. The prevalence [1.6% (2/124)] of HBsAg in adults, aged 21 to 29 years, was considerably lower than that [4.1% (10/246)] observed in adults older than 29 years of age. A significant increase in prevalence of HB infection was observed with increase in age (*P* = 0.0002, chi-squared test).

**Table 2:** **Prevalence of hepatitis markers among the participants grouped according to their age**

**Age group**	**HBsAg**			**anti-HBs**			**anti-HBc**		
	**No. positive/no.tested**	**%**	**95%CI**	**No. positive/no.tested**	**%**	**95%CI**	**No. positive/no.tested**	**%**	**95%CI**
Children									
6 mos – <5 yrs	0/432	0.0%	0.0-1.1	424/432	98.1%	96.2-99.1	0/432	0.0%	0.0-1.1
Adolescent									
16-20 yrs	7/124	5.6%	2.3-11.3	22/124	17.7%	11.5-25.6	11/124	8.9%	4.5-15.3
Adult (n = 370)									
21-29 yrs	2/124	1.6%	0.2-5.7	34/124	27.4%	19.8-36.2	22/124	17.7%	11.5-25.6
30-39 yrs	5/123	4.1%	1.3-9.2	39/123	31.7%	23.6-40.7	27/123	22.0%	15.0-30.3
40-49 yrs	5/123	4.1%	1.3-9.2	34/123	27.6%	20.0-36.4	26/123	21.1%	14.3-29.4
Total	19/926	2.0%	1.3-3.2	553/926	59.7%	56.5-62.9	86/926	9.2%	7.5-11.3

The rate of anti-HBs seropositivity was 98.1% (424/432) in children, 17.7% (22/124) in adolescents, and 28.9% (107/370) in adults (Table 2). All 432 children were anti-HBc negative. The rate of anti-HBc seropositivity was 8.9%, 17.7%, 22.0%, and 21.1% for those aged 16–20, 21–29, 30–39 and 40–49, respectively. The rate of anti-HBc seropositivity was 17.4% (86/494) for adolescents and adults, and 9.2% (86/926) overall.

The combined results for the detection of the three serologic markers for HB infection used in this study showed that 424 out of 432 (98.1%) children, 15 out of 124 (12.1%) adolescents and 59 out of 370 adults (15.9%) were positive for anti-HBs but not for the other two markers and thus can be considered immune to HBV infection due to vaccination (Table 3). The total number of adolescents and of adults positive to anti-HBS was actually 22 (17.7%) and 105 (28.4%), respectively, if those positive to anti-HBc were included (Table 3). Eight children (1.9%) were negative to all the tests in spite of the fact that they received a complete series of HB immunization (Table 3). There were 94 adolescents (75%) and 232 adults (62.7%) who were negative to all the serological tests (Table 3).

**Table 3:** **Number of children, adolescents and adults according to the combination of results for the detection of serologic markers for HB infection and interpretation of results**

	**Serologic markers**			**Children(6 mos- < 5 yrs)**	**Adolescents(16–20 yrs)**	**Adults(21–49 yrs)**	**Adolescents + Adults(16–49 yrs)**	
Combination	HBsAg	anti-HBc	anti-HBs					Interpretation [1,2]
1	-	-	-	8*	94	232	326	susceptible, never infected
2	+	-	-	0	4	4	8	early acute infection
3	+	+	-	0	3	6	9	acute or chronic infection
4	-	+	-	0	1	21	22	past infection or false positive (i.e. susceptible), or “low level” chronic infection
5	-	+	+	0	7	46	53	Past infection; recovered and immune
6	+	-	+	0	0	0	0	-------
7	-	-	+	424	15	59	74	Immune due to hepatitis B vaccine
8	+	+	+	0	0	2	2	Acute or chronic infection
Total				432	124	370	494	

*Received complete series of immunization.

Prevalence rates of HBsAg among adult population showed geographic variations with significantly higher prevalence (P = 0.0081) in the urban Central Division (Table 4). Significantly higher prevalence of HBsAg positivity (*P* = 0.03, chi-squared test) was observed in female participants in this survey (Table 4). A higher immunity level by vaccination was seen in the Northern Division but this was not significant. Among the different ethnic groups, Fijians had significantly the highest rate (*P* = 0.0041, chi-squared test) of HB infection (Table 4).

**Table 4:** **Prevalence of hepatitis markers among the adult residents classified based on locality, gender and ethnic grouping**

	**HBsAg**			**anti-HBs**			**anti-HBc**		
	**No. positive/no.tested**	**%**	**95%CI**	**No. positive/no.tested**	**%**	**95%CI**	**No. positive/no.tested**	**%**	**95%CI**
**21-49 years**	12/370	3.2%	1.8-5.8	107/370	28.9%	24.4-33.9	75/370	20.3%	16.4-24.8
**Division**									
Central	10/149	6.7%	3.3-12.0	50/149	33.6%	26.0-41.7	35/149	23.5%	16.9-31.1
Western	1/148	0.7%	0.0-3.7	17/148	11.5%	6.8-17.8%	19/148	12.8%	7.9-19.3
Northern	1/73	1.4%	0.0-7.4	40/73	54.8%	42.7-66.5	21/73	28.8%	18.8-40.6
**Gender**									
Male	7/310	2.3%	1.0-4.8	88/310	28.4%	23.5-33.8	63/310	20.3%	16.1-25.3
Female	5/60	8.3%	2.8-18.4	19/60	31.7%	20.3-45.0	12/60	20.0%	10.8-32.3
**Ethnic group**									
Fijian	9/137	6.6%	3.0-12.1	73/137	53.3%	44.6-61.9	50/137	36.5%	28.4-45.1
Indian	1/204	0.5%	0.0-2.7	20/204	9.8%	6.1-14.7	15/204	7.4%	4.2-11.8
Others	2/29	6.9%	0.8-22.8	14/29	48.3%	29.4-67.5	10/29	34.5%	17.9-54.3

### Hepatitis B immunization coverage

Immunization coverage rate in this study was defined as the percentage of children who received at least three doses of HB vaccine. There were 439 children whose immunization data were available for review (Table 5). The immunization coverage based on child health card was very high in the three divisions (>98% per division; total average of 99.3%). A total of 379 (86.3%) children received the first dose (“birth dose”) of HB vaccine within 2 days from birth. WHO`s recommendation is that the first dose of hepatitis B vaccine be given within 24 hours of birth [9].

**Table 5:** **Coverage and timeliness of hepatitis B immunization in children from each health division**

**Category**	**No. of children with the feature in each category/total no. of children (%)**			
	**Central**	**Western**	**Northern**	**Total**
Coverage in terms of number of dose (s) received during hepatitis B immunization (total n = 439)				
Three doses or more	191/194 (98.5)	173/173 (100.0)	72/72 (100.0)	436/439 (99.3)
Only two doses	3/194 (1.5)	0/173 (0.0)	0/72 (0.0)	3/439 (0.7)
Only one dose	0/194 (0.0)	0/173 (0.0)	0/72 (0.0)	0/439 (0.0)
Timeliness of birth dose (total n = 439)				
Within 2 days	170/194 (87.6)	156/173 (90.2)	53/72 (73.6)	379/439 (86.3)
Day 3 – 10	16/194 (8.2)	8/173 (4.6)	17/72 (23.6)	41/439 (9.3)
Day 10 – 30	5/194 (2.6)	5/173 (2.9)	1/72 (1.4)	11/439 (2.5)
Never immunized	3/194 (1.5)	4/173 (2.3)	1/72 (1.4)	8/439 (1.8)
Timeliness of completion of 3 doses of HBV in children < 2 years				
(total n = 176)				
On – schedule	46/62 (74.2)	50/79 (63.3)	7/35 (20.0)	103/176 (58.5)
Delayed	16/62 (25.8)	29/79 (36.7)	28/35 (80.0)	73/176 (41.5)

“On-schedule” for immunization was defined as giving a one month leeway after due date of third HB vaccine dose to explore timeliness of HBV immunization [10]. This definition was applied to children between 6 months to < 2 years of age and born after the change of previous Fiji’s HBV standard immunization schedule for 3 doses to the present schedule for four doses of vaccine given at birth, 6, 10 and 14 weeks [5,6,8]. The result shows that 58.5% of children were on-schedule overall (Table 5).

## Discussion

The HBV immunization program has been in place in Fiji since the 1990s and this study is timely with a hypothesis that prevalence of HBV infection should be negligible or low among the children. All 432 children were negative to both HBsAg and anti-HBc and this adds some support to this hypothesis. However, a few (8/432) were negative to anti-HBs or did not form antibody after completing 3 doses of HB vaccination on schedule. The non-seroconversion of antibody against HBV or the natural decline in the level of the antibodies to undetectable level (<10 mIU/mL) through time is a possible explanation [11,12]. Studies have shown that anti-HBs concentrations declines rapidly within the first year and more slowly thereafter. However, despite the possible decline to less than detectable level, there remain some levels of protection against HBV infection due to immune memory [13,14].

The prevalence of HBsAg among children immunized is less (0.0%) compared to 0.7% in 1998 (Table 6), suggesting the effectiveness of the HB vaccine and effectiveness of the childhood immunization program in the country. A prevalence of 3.2% of HBsAg and 20.3% of anti-HBc was found in the adult population. The exclusion of a number of previous high-risk blood donors with HBV from the survey, confirms the circulation of the hepatitis B infection in the adult population [15]. A higher prevalence of HB infection among the adult population was observed in the Central Division. A possible explanation could be that large urban population has the largest segment of uninformed personnel and other risk occupational groups for sexually transmitted diseases.

**Table 6:** **Comparison of test results of hepatitis B markers surveyed in 1998, 2008 and 2009**

	**Immunized children**		**Unimmunized adult**	
	**1998** ^**a**^	**2008**	**1998** ^**b**^	**2009** ^**c**^
HBsAg positive	2/285 (0.7%)	0/432 (0.0%)	19/290 (6.6%)	12/370 (3.2%)
anti-HBs positive	217/282 (77.0%)	424/432 (98.1%)	168/290 (57.9%)	107/370 (28.9%)
anti-HBc positive	15/281 (5.3%)	0/432 (0.0%)	195/290 (67.2%)	75/370 (20.3%)

^a^1998 survey results were for children (<6 years old) who were immunized with hepatitis B vaccine [8].

^b^Unimmunized adults surveyed in 1998 were mothers who were born before hepatitis B vaccine was incorporated in the child hood immunization program [8].

^c^Unimmunized adults surveyed in 2009 were from this present study; their ages were from 21 to 49 years and they were born before hepatitis B vaccine was incorporated in the childhood immunization program.

Two hundred thirty two of the adult participants (62.7%) and 94 (75%) of adolescents had never been infected with HBV and were not immune (i.e. they were susceptible) to HBV infection (HBsAg, anti-HBs and anti-HBc negative). Current treatment of HBV infection is not highly effective, therefore, HB immunization is recommended for the above unimmunized populations [1,16]. There is a need to strengthen efforts to reach this susceptible populations with HBV vaccination. The offer of HBV immunization to this age group in institutions should be strengthened. These institutions should not be limited to health care facilities, hostels, boarding schools, blood banks, etc. Secondly, the Ministry of Health should actively advocate HBV vaccination in its sexual reproductive health and adolescence health programs.

From among the 494 adolescents and adults, 86 (17.4%) had evidence of past HBV infection (anti-HBc positive) for which 75 (87.2%) did not develop chronic infection (anti-HBc positive but HBsAg negative) but a remaining 11 (12.8%) became chronic carriers (anti-HBc and HBsAg positive). These results are also observed in other studies with a few identified as carriers while others in complete recovery [12,17].

The test results showed 22 (4.5%) adolescents and adults were anti-HBc positive but negative to the other two markers for HBV infection. Although, a typical interpretation is a history of past HBV infection, it is also possible that it was a false positive result (i.e. susceptible) or the participant had a “low level” chronic infection [1,2].

Although the criteria for the sample population are different from 1998, this study suggests a possible decline in the prevalence of HBsAg positives and anti-HBc negatives among immunized children and unimmunized adults (Table 6). The prevalence of HBsAg among unimmunized population has decreased from 6.6% in 1998 [8], surveyed among mothers representing unimmunized population, to 3.2% in this survey. The reasons for this decrease are unknown, however, it may be attributed to the increased awareness on HBV infection and its prevention in addition to the increased uptake of HBV vaccination in the population.

HBV immunization coverage found in this study was much higher than that reported in 2007 (Table 7) [18]. Reported coverage may be influenced by overestimated denominators, target population, and underestimated numerators based on inaccurate or delayed submission of monthly reports on the number of vaccine doses administered. However, coverage data are often verified with regular coverage surveys.

**Table 7:** **Comparison of the immunization coverage in children for 2007 and for the present study in the three health divisions**

	**% coverage for 2007** [18] **(% coverage for the present study)**			
	**Central**	**Western**	**Northern**	**Total**
Birth dose	67.4 (98.5)	92.0 (97.7)	89.3 (98.6)	80.3 (98.2)
Third dose^a^	76.3 (98.5)	88.0 (100.0)	83.6 (100.0)	81.9 (99.3)

^a^Third dose of hepatitis B immunization was given as DTP-HepB-Hib.

In terms of prevention of HBV, preventing infections acquired at birth and in early childhood is critical. The key to reduce mother-to-child transmission is timely administration of the first dose of the Hepatitis B vaccine within 24 hours of birth. Efficacy of the vaccine in preventing perinatal transmission declines with increasing intervals between birth and the time of administration of the vaccine [9].

The overall, immunization coverage for the first dose of HB vaccine at birth in this study was very good at 98%. However, timeliness of the first dose administration remains a challenge. About 15% of children received the first dose of HB vaccine unacceptably late.

A trend of lower proportion for both timely administration of first HB immunization (*P* = 0.0009, chi-squared test) and “on-schedule” immunization in Northern Division were observed. Delayed immunization is not a major problem if the child completes a series of HB immunization however, timeliness of HB birth dose should be ensured.

There are several limitations to this study. The time and travel constraints could not accommodate the samples from remote outer islands. This survey could not yield information on the rate of HBV infections in children who were born to HBsAg positive mothers as participating children were randomly selected. Despite its limitations, this study is the first to describe the prevalence of HBV infection in different age groups. The results will help public health officials in Fiji in their plan for strengthening programmatic actions for hepatitis B control.

## Conclusion

This survey suggests a successful HB immunization program in Fiji. The program that has been in place since 1989 has led to a further reduction of HBV infection rates in the population and increase of immunity levels among children since the introduction of the program.

## Methods

### Study setting, immunization records and sample collection

A serologic survey was conducted from three out of the four health service divisions in Fiji namely Central, Western, and Northern Divisions. Participants from the Eastern Division, were excluded from this survey due to distance and time constraints. Two different survey settings were followed. In the first setting, a total of 450 healthy children aged 6 months to <5 years were recruited in the three health service divisions from September to October 2008 (Figure 1 and Table 1). A sampling size of 450 was calculated based on the estimated population per the target age group using the cluster survey method. These children were likely vaccinated with HBV at birth as part of the national HB immunization program. Zone nurses at the three selected divisions facilitated child listing exercise to ensure that the target numbers per division were proportionate. Blood collection for the survey was done at the health centers. The health record card of each child was reviewed to calculate HB immunization coverage rates and describe any association between immunization status and serologic tests. In the second setting, a total of 125 healthy adolescents (16–20 years of age) born after the launch of the national HB immunization program and a total of 375 adults (21–49 years of age) born before the launch were recruited in the three divisions from May to July 2009. For determining the sampling size of adolescent and adults, time, travel and financial constraints were considered. Thus 100 target samples (25 adolescents and 75 adults) at Northern Division (Labasa area), and 200 target samples (50 adolescents and 150 adults) each at Central Division (Suva) and Western Division (Lautoka) were found practical and less expensive and valid for the representative estimates for three different divisions and two different age categories (Table 1). These individuals were recruited from the blood bank services and only the blood samples they donated for the first time were included. Succeeding blood samples from repeat safe donors (those safe donors who donated blood repeatedly) and all blood samples from high risk donors (e.g. recognizable person with HBV infection, sex contacts of person with HBV infection) were excluded. The survey on adolescents and adults provided additional information on the extent of hepatitis B infection in the population. Blood samples were collected after obtaining a written consent from adult participants and parents of enrolled minors. Convenience sampling of the adult population from the blood banks was done in a manner that would ensure blood samples were proportionally representative of the different age groups.

### Ethical approval

The National Health Research Committee (NHRC) of Fiji provided ethical approvals for the survey on children on 11 September 2008 (FNRERC Reference Number: 2008–026) and for adolescents and adults on 11February 2009 (FNRERC Reference Number: 2009–002).

### Serologic tests

A sample of at least 5 ml of venous blood was obtained from each participant and sample was centrifuged on the day of collection. The serum samples were then transported on ice to the National Public Health Laboratory of Mataika house (Fiji Centre for Communicable Disease Control) in Suva, Fiji. Serological tests for children samples were conducted at Mataika house, FFCDC while adolescent and adults samples were tested at the Department of Virology, Institute of Tropical Medicine at Nagasaki University in Japan. HBsAg, anti-HBs and anti-HBc were detected by Reverse Passive Hemagglutination (RPHA) (Mycel II HBsAg; Institute of Immunology, Co., Ltd, Tokyo, Japan), Passive Hemagglutination (PHA) (Mycell II anti-HBs; Institute of Immunology Co., Ltd, Tokyo, Japan), and PHA (Mycell anti-rHBc) methods respectively. The samples further underwent confirmatory testing for anti-HBC by enzyme-linked immunosorbent assay (ELISA) kit (DRG®Anti-Hbc ELISA, DRG International, Inc. USA).

A positive result for HBsAg, for anti-HBs, and for anti-HBc was interpreted as the participant having ongoing or chronic infection with HBV, having immunity from HBV infection, and having past HBV infection, respectively [1,16].

### Analysis of data

All the epidemiological and laboratory data were analyzed blindly in Epi info Version 3.3.2. A chi-square test was applied with a p < 0.05 level of significance.
